# Effect of Double Transition Metal Salt Catalyst on Fushun Oil Shale Pyrolysis

**DOI:** 10.1155/2020/6685299

**Published:** 2020-11-04

**Authors:** Xiaoyang Liu, Haodan Pan, Chuang Guo, Xiaojing Di, Hongxiang Hu

**Affiliations:** ^1^College of Petroleum Engineering, Liaoning Shihua University, Fushun 113301, China; ^2^Beijing Gas Energy Development Co. Ltd, Beijing 100000, China; ^3^CAS Key Laboratory of Nuclear Materials and Safety Assessment, Institute of Metal Research, Chinese Academy of Sciences, Shenyang 110016, China

## Abstract

Shale ash (SA) as the carrier, the ratio of Cu to Ni in the Cu-Ni transition metal salt being, respectively, 1 : 0, 2 : 1, 1 : 1, 1 : 2, 0 : 1, the double transition metal salt catalyst (Cu_m_Ni_n_/SA) was prepared to explore the effect of such catalysts on the pyrolysis behavior and characteristics of Fushun OS. The research results show that the temperature (*T*_max_) corresponding to the maximum weight loss rate decreased by 12.9°C, 4.0°C, and 3.6°C; and the apparent activation energy decreased by 35.2%, 33.9%, and 29.6%, respectively, after adding catalysts Cu_0_Ni_1_/SA in pyrolysis. The addition of Cu_0_Ni_1_/SA and Cu_2_Ni_1_/SA further improves the shale oil (SO) yield of 3.5% and 3.1%, respectively. Cu_0_Ni_1_/SA produces more aromatic hydrocarbons, which, however, weakens the stability of SO and is of toxicity in use. After analyzing the pyrolysis product—semicoke (SC) and SO—with ATR-FTIR and GC-MS methods, Cu_m_Ni_n_/SA promotes the secondary cracking and aromatization of OS pyrolysis, increasing the content of the compound of olefins and aromatics in SO, and hastening the decomposition of long-chain aliphatic hydrocarbons to short-chain aliphatic hydrocarbons.

## 1. Introduction

With the shortage of traditional energy resources against the increasing oil demand, oil shale is considered to be a type of ideal alternative energy source to substitute oil owing to its abundant reserves. There are approximately 68.92 billion tons shale oil converted from proven oil shale in the world, which is three times the amount of crude oil reserves [1] and approximately accounts for 35% of the total global energy [2]. China is abundant in oil shale reserves, and the oil content is above the average. Among them, oil shale reserves with an oil content of more than 5% to 10% are 266.435 billion tons, and more than 10% are 126.694 billion tons [3]. Liaoning Fushun mining area, the third-largest mining area in the country, boosts approximately 3.6 billion tons of the oil shale reserves [4].

Shale oil and shale gas can be obtained through the pyrolysis of oil shale (OS). Catalytic pyrolysis increases the conversion rate of oil shale pyrolysis and the yield of shale oil as well as its quality [5, 6]. Domestic and foreign scholars have done extensive experimental studies on the catalytic pyrolysis of oil shale with different catalysts. Gai et al. [7] studied the influence of the presence of pyrite on the pyrolysis behavior of oil shale. The iron contained in pyrite positively affects the pyrolysis behavior of oil shale and promotes its volatilization, thus increasing the yield of liquid and gas products. Cao [8] introduced a type of catalyst from oil shale rock as raw material to obtain light feedstock oil, which not only greatly reduces the cost of producing light fuel oil but also increases the oil quality by adding the catalyst. Williams et al. [9, 10] studied the effect of the ZSM-5 zeolite catalyst on the pyrolysis of oil shale in Kark, Pakistan. The addition of catalyst increases the content of gas products and shale semicoke, reducing the yield of shale oil, promoting the conversion of long-chain alkanes and olefins into low-molecular-weight and short-chain alkanes, and decreasing the total nitrogen and sulfur content in the catalyzed oil. Wang et al. [11] added alkali metal carbonates K_2_CO_3_, MgCO_3_, Na_2_CO_3_, and CaCO_3_ to oil shale, and the results showed that different alkali metal carbonates had different catalytic effects on oil shale. MgCO_3_ has an obvious effect compared with other alkali metal carbonates, and it is especially violent at the beginning of pyrolysis. Jiang et al. [12] and Pulushev et al. [13] studied the effects of pyrolysis conditions and transition metals on the pyrolysis products and characteristics of Huadian oil shale, finding that the addition of transition metal cobalt salts to OS increases the selectivity of aromatics and promotes the aromatization of olefins. Chang et al. [14] studied the effects of FeCl_2_·4H_2_O, CoCl_2_·6H_2_O, NiCl_2_·6H_2_O, and ZnCl_2_ on the pyrolysis of OS, summarizing that all these four metal salts enhance the secondary cracking of shale oil, reduce oil production, and improve the pyrolysis gas production. All these studies affirm that catalyst increases either the pyrolysis conversion rate or product yield. However, there are relatively few reports on the OS pyrolysis catalyzed by shale ash (SA) with double transition metal salts as the carrier is relatively few.

In this paper, shale ash (SA) as the carrier, the ratio of Cu to Ni in Cu-Ni transition metal salt-containing being different, the double transition metal salt catalyst (Cu_m_Ni_n_/SA) was prepared to explore the effect of such catalysts on the pyrolysis characteristics of Fushun OS. The thermogravimetric method was applied to analyze the effect of the double transition metal catalysts containing different ratios Cu to Ni in Cu_m_Ni_n_/SA groups on the pyrolysis characteristics of OS. The components of pyrolysis product shale oil (SO) were analyzed by ATR-FTIR and GC-MS, while the effects of the catalyst on the activation energy of OS pyrolysis were analyzed by the Coats Redfern model.

## 2. Experimental Materials and Methods

### 2.1. Materials

OS and SA were obtained from Fushun, Liaoning Province, China. OS and SA samples were first crushed and screened into a particle size of 10-18 mesh and 40-60 mesh before the experiment, and then cleaned with deionized water for 7-8 times, and eventually dried overnight in a blast drying oven at 80°C. The main properties of OS and SA samples are shown in Table 1.

**Figure 1:**
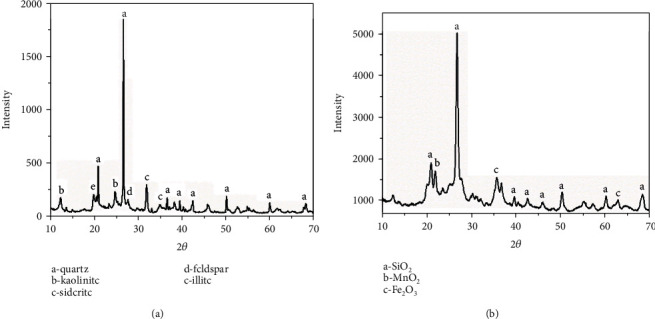
XRD patterns of oil shale (OS) and shale ash (SA): (a) OS; (b) SA.

The inorganic crystalline phases in OS and SA were analyzed by X-ray diffraction (XRD), and the results are shown in Figure 1. The XRD spectrum shows that the minerals in OS are mainly composed of quartz and aluminosilicates including kaolinite and illite, and also a small amount of carbonate. The specific components are shown in Table 2.

**Figure 2:**
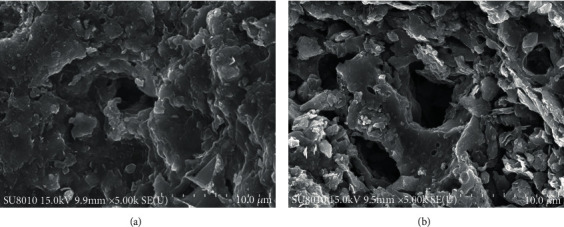
Scanning electron microscopy (SEM) of OS and SA: (a) OS; (b) SA.

Figure 2 is the scanning electron microscopy (SEM) of OS and SA, presenting the maldistribution and irregular but the certain pored structure of the mineral particle size in the OS, which belongs to a type of solid sedimentary rocks composed of the scaly-structured clay kaolinite and mica mineral.  Figure 2(b) clearly shows the larger pored structure of the SA compared with that of OS, enabling the SA to load transition metal salt as a carrier.

The equal volume impregnation method was applied to prepare for the catalyst samples. First, the water absorption of the carrier SA was measured. The loading amount of the transition metal salt was set as 3 wt.% for the distinct experiment statistics (the ratio of the mass of the two types of transition metal salts to the sum of the mass of the two types of transition metal salts and SA was 3 wt.%). The transition metal salt (CuCl_2_·2H_2_O, NiCl_2_·6H_2_O) was accurately weighed with different Cu/Ni ratios (1 : 0, 2 : 1, 1 : 1, 1 : 2, 0 : 1) and then dissolved in the same volume of deionized water. Mechanically stirred and mixed with a glass rod, SA was added into the solution and again fully stirred and immersed for 12 hours. After a 20-hour forced air drying at 130°C, the Cu-Ni/shale ash-based double transition metal catalyst was obtained and stored in seal preservation for later use.

The prepared SA-grouped double transition metal catalyst is named as Cu_m_Ni_n_/SA (*m* for the mass fraction ratio of Cu metal salt and *n* for the Ni metal salt in the catalyst), and the sample of Cu_m_Ni_n_/SA mixed with OS is expressed as OS-Cu_m_Ni_n_/SA, for example, OS-Cu_1_Ni_1_/SA represents SA the mixed pyrolysis with a load mass fraction ratio of OS to Cu-Ni of 1 : 1.

**Figure 3:**
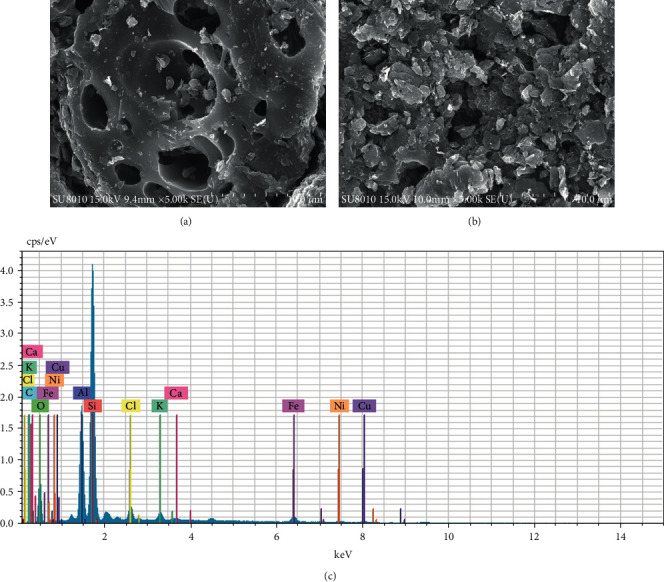
SEM/EDS analysis image of Cu_m_Ni_n_/SA. (a) Scanning electron micrograph (SEM) before SA loading metal salts. (b) SEM after SA loading metal salts. (c) Energy dispersion spectrometer (EDS) image after SA loading metal salts.

The morphology analysis of SA after loading metal salt is shown in Figure 3. Compared with the initial SA, the surface of Cu_m_Ni_n_/SA is covered with scaly material, and the pores become smaller and shallower (Figure 3(a)). Through SEM/EDS analysis, the material composition in the pores contains Ni and Cu elements, indicating that the metal salt has been loaded into the pores of SA through the impregnation method (Figure 3(b)).

### 2.2. Laboratory Apparatus

The Nicolet iS50 model Fourier transform attenuated total reflection infrared spectroscopy (ATR-FTIR) was applied to analyze the characteristics of OS and SO, and Netzsch STA 449 F5 thermogravimetric analyzer-mass spectrometry (TG-MS) to study OS thermal weight loss behavior and component. The SO obtained from the experimental pyrolysis was extracted with dichloromethane, and the SO component was analyzed with the GC-MS (Agilent 7890A/5975C from NYSE: A, United States). The ATR-FTIR spectrum was recorded between 4000 cm^−1^ and 400 cm^−1^, and the spectral resolution was 4 cm^−1^. The sensitivity of the microbalance for TG detection was less than ±0.1 *μ*g, and the temperature accuracy was ±0.5°C. In addition, in order to avoid the limitation of heating transfer, a blank sample was used before the experiment to baseline the influence of the buoyancy and weight loss of the crucible on the experimental data.

### 2.3. Experimental Methods

First, a certain amount of mixed sample (10 ± 0.5 mg) of OS and SA-grouped transition metal salt catalyst was weighed and placed into a thermogravity crucible with a height of 4 mm and a diameter of 6 mm. The 50 mL/min argon was used as the purge gas, and the 30 mL/min of argon as the protective gas. Then, the temperature was lifted from room temperature to 900°C at a heating rate of 10°C/min. The mixing ratio of OS to catalyst was 2 : 1. The gas produced during the pyrolysis was purged into the mass spectrometer through the capillary column connecting to the thermogravimetric analyzer. Besides, the connecting tube between the thermogravimetry and the mass spectrometer was heated to 255°C to prevent the gas from condensing in the capillary.

The weight loss rate of pure OS is calculated by formula (1):

(1) $$\begin{array}{c} M_{\text{OS}}=M_{\text{Loss}}/r_{\text{OS}}. \end{array}$$

*M*_Loss_ is the weight loss rate of OS during the mixed pyrolysis of transition metal salt and OS, and *r*_OS_ is the mass percentage of OS in the sample.

### 2.4. Kinetics Analysis

Assuming *m*_0_ represents the initial quality of the oil shale. It was heated according to the preset heating program where it underwent the thermal decomposition reaction. The pyrolysis conversion rate of OS *α* can be expressed as (*t* time for the time, *m*_*t*_ for the mass of the sample, and *m*_∞_ for the final mass of the residue unable to be decomposed)

(2) $$\begin{array}{c} \alpha =\frac{m_{0}-m_{t}}{m_{0}-m_{\infty }}. \end{array}$$

The decomposition rate in the decomposition reaction can be expressed as

(3) $$\begin{array}{c} \frac{d\alpha }{dt}=kf\left(\alpha \right). \end{array}$$

In this formula, *k* is the Arrhenius rate constant, *k* = *A*exp(−*E*/*RT*); *A* is the prefactor, (S^−1^); *E* is the activation energy, (kJ/mol); *T* is the thermodynamic temperature, (K); *R* is the gas constant, in units of J/(mol·K). The overall reaction equation of OS pyrolysis can be expressed as

(4) $$\begin{array}{c} \frac{d\alpha }{dt}=A\exp\left(-\frac{E}{RT}\right)f\left(\alpha \right). \end{array}$$

In nonisothermal conditions, the heating rate can be expressed as *β* = *dT*/*dt*; then, formula (3) can be rewritten as

(5) $$\begin{array}{c} \frac{d\alpha }{dT}=\frac{A}{\beta }\times \exp\left(-\frac{E}{RT}\right)f\left(\alpha \right). \end{array}$$

The functional form of *f*(*α*) and its reaction order is determined by the reaction type or mechanism, normally *f*(*α*) = (1 − *α*)^n^.

## 3. Experimental Results and Discussion

### 3.1. Effect of Transition Metal Salt Catalysts on OS Pyrolysis

#### 3.1.1. Analysis of Catalytic Cracking Behavior

**Figure 4:**
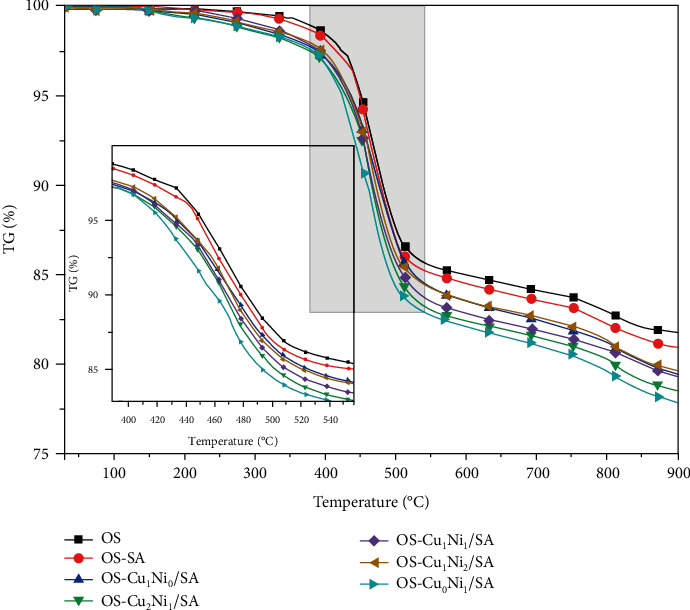
TG curves of mixed pyrolysis of OS with different Cu_m_Ni_n_/SA.

**Figure 5:**
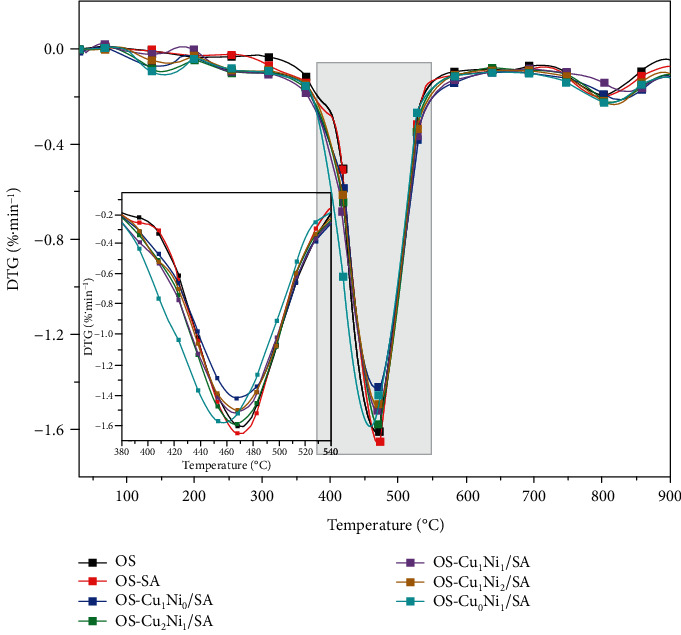
DTG curves of mixed pyrolysis of OS with different Cu_m_Ni_n_/SA.

Figures 4 and 5 show the thermogravimetric (TG, DTG) curves of OS after adding different transition metal salts. The quality loss of OS involves various chemical and physical processes and can be divided into three following stages [15, 16]: the figures below show that the mass loss in stage one (<200°C), mainly caused by the evaporation of water, especially the adsorbed water from clay minerals and interlayer water, was relatively small due to the sample's lower moisture content after the blast drying procedure. Stage two (200-600°C) was the decomposition of organic matter. The kerogen molecules and other organic components in the OS were decomposed and produce asphaltenes, which then continued to decompose into various small aromatic molecules or aliphatic molecules. Technically, this stage can also be considered as the oil production stage in OS pyrolysis [12], where a series of complex reactions including ring-opening reaction, depolymerization reaction, and repolymerization reaction [17, 18] would occur when the temperature rose to a certain level. These reactions promoted the addition and rearrangement of organic matter in the OS and the production of different types of organic compounds. The rapid pyrolysis occurred between 417.2 and 519.9°C, and the weight loss rate in this stage reached as high as 13.58 wt.%, accounting for 65.35 wt.% of the total weight loss. The maximum weight loss peak also appeared. In the third stage (>600°C), the decomposition of organic matter basically finished and occurred mainly the decomposition reaction of inorganic minerals (such as carbonate and clay minerals).

The figure suggests a similar trend of the curves of OS TG and DTG after adding Cu_m_Ni_n_/SA. The TG curves moved left to the low-temperature zone after adding SA and different Cu_m_Ni_n_/SA to the direct OS pyrolysis, manifesting that the temperature of OS after adding SA and Cu_m_Ni_n_/SA was lower than that of the direct OS pyrolysis under the same weight loss rate in its main pyrolysis stage. The violate evolution content increased and the final coke output decreased to a certain extent. The catalytic effect of Cu_m_Ni_n_/SA was significantly higher than that of SA, among which OS-Cu_0_Ni_1_/SA had the minimum coke output, and the total mass loss rate in descending order was OS − Cu_0_Ni_1_/SA < OS − Cu_2_Ni_1_/SA < OS − Cu_1_Ni_1_/SA < OS − Cu_1_Ni_0_/SA < OS − Cu_1_Ni_2_/SA < OS − SA < OS. The DTG curves show three sharp weight loss peaks during the pyrolysis of OS, which, respectively, corresponds to the decomposition of moisture (90~200°C), organic matter (200~600°C), and inorganic minerals (700~900°C). The first maximum weight loss rate in the second stage was significantly higher than that in the first and third stages, indicating that the mass-loss rate in the second stage was faster than that in the first and third stages.

Combining with Table 3, it can be seen the addition of SA and Cu_m_Ni_n_/SA to varying degrees lowered the temperature (*T*_max_) reaching the occurrence of maximum mass loss rate. The decrease in temperature corroborates that SA and Cu_m_Ni_n_/SA can catalyze the cracking reaction to a certain extent and further activate the release process of volatile substances. Within an appropriate temperature range, the presence of transition metal salt ions accelerates the cleavage of branched functional groups in kerogen and promotes the formation of cracked oil gas phase, thereby lowering the reaction temperature. Among them, the *T*_max_ of OS-Cu_0_Ni_1_/SA had the sharpest decline of 2.74% compared with that of the direct OS pyrolysis. The *T*_max_ of each sample in descending order was ranked as OS − Cu_0_Ni_1_/SA < OS − Cu_2_Ni_1_/SA < OS − Cu_1_Ni_1_/SA < OS − Cu_1_Ni_2_/SA < OS − Cu_1_Ni_0_/SA < OS − SA. Moreover, the addition of Cu_m_Ni_n_/SA lowered the start and end temperatures in the main pyrolysis stage, as well as the maximum weight loss rate to a certain extent, but expanded the devolatilization temperature range. It can also be seen from Table 3 that the samples adding SA and Cu_m_Ni_n_/SA had a greater weight loss rate within all temperature ranges, indicating that SA and Cu_m_Ni_n_/SA promotes the pyrolysis reaction of OS, including the decomposition of inorganic minerals. Thermal cracking behavior confirms that OS-Cu_0_NiA, OS-Cu_2_Ni_1_/SA, and OS-Cu_1_Ni_1_/SA have better catalytic effects.

#### 3.1.2. Kinetics Analysis

Kinetic analysis, combined with the macroscopic phenomenon of the reaction, reflects the relationship between the energy and the movement of substances and reveals the pyrolysis reaction mechanism so as to control the chemical reaction. Among various analysis models, the Coats-Redfern (C-R) method has been used to calculate the relevant kinetic parameters of solid fuel pyrolysis [19, 20]. This method was applied to the thermal kinetic calculations in this section to analyze the kinetics in the main pyrolysis stages. After the analysis of the kinetic parameters of the samples adding different Cu_m_Ni_n_/SA, the values of the thermokinetic parameters *E*, *A*, and *R*^2^ obtained are shown in Table 4.

Table 4 shows that SA and Cu_m_Ni_n_/SA reduce the activation energy required for the pyrolysis reaction in the main pyrolysis stage. OS forms asphaltenes in the second stage and then continues to be decomposed and produce volatile substances. A certain amount of energy needs to be absorbed to support the occurrence of related depolymerization cracking reactions in this process. It can be seen from Table 4 that the direct OS pyrolysis needs to absorb approximately 39.2 kJ/mol for the related chemical reactions. Adding SA and Cu_m_Ni_n_/SA, the apparent activation energy-reduced, and the reduction degree of Cu_m_Ni_n_/SA on activation energy was significantly higher than that of SA. Among them, OS-Cu_0_Ni_1_/SA reduced the maximum activation energy of 13.8 kJ/mol, accounting for 35.2% of the activation energy required for OS pyrolysis; OS-Cu_2_Ni_1_/SA is the second and required 13.3 kJ/mol activation energy to reduce pyrolysis, accounting for 33.9% of the activation energy required for the OS pyrolysis. Scholars also believe that metal cations, as a strong polar core, may be embedded into the crystal lattice of macromolecules after interacting with kerogen and other macromolecules, thus deforming the electronic structure on the surface and causing a dynamic induction effect. Such an effect reduces the bond energy of the C-C bond, requiring less energy for the reaction, and leading to a decrease of the activation energy. From the kinetic analysis, the order of the samples that reduced the activation energy of the reaction was OS − Cou_0_Ni_1_/SA > OS − Cu_2_Ni_1_/SA > OS − Cu_1_Ni_0_/SA > OS − Cu_1_Ni_1_/SA > OS − Cu_1_Ni_2_/SA > OS − SA.

According to the fitting of the C-R model, reactions of the rapid pyrolysis stage of OS belong to the first-order reaction, with a correlation coefficient *R*^2^ of above 0.98. Such a high value proves the reliability of this calculation model. The change of the index factor also demonstrates that Cu_m_Ni_n_/SA might reduce the number of collisions between material particles per unit time. Table 4 shows that the catalytic effect of different samples: OS-Cu_0_Ni_1_/SA (25.4 kJ/mol) was stronger than OS-Cu_1_Ni_0_/SA (26.3 kJ/mol), that is, NiCl_2_·6H_2_O performed better than CuCl_2_·2H_2_O, but OS-Cu_2_Ni_1_/SA lied in the middle. However, OS-Cu_1_Ni_1_/SA and OS-Cu_1_Ni_2_/SA were relatively poor, and Cu_m_Ni_n_/SA did not increase due to the higher loading of NiCl_2_·6H_2_O. Therefore the two transition metal salts supported by Cu_m_Ni_n_/SA were complicated in the OS pyrolysis process, rather than catalyzing the OS pyrolysis reaction in their respective ranges.

### 3.2. Effect of Catalyst on the Output of OS Pyrolysis Products

**Figure 6:**
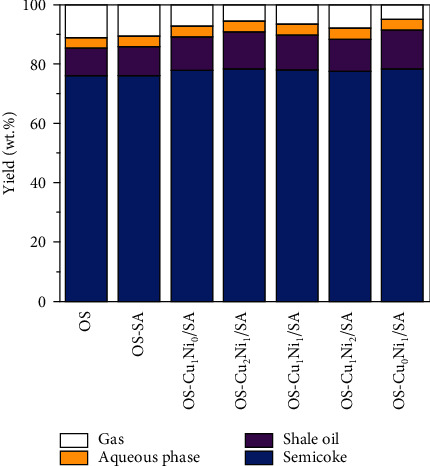
The average yield of the catalytic pyrolysis products of OS.

**Figure 7:**
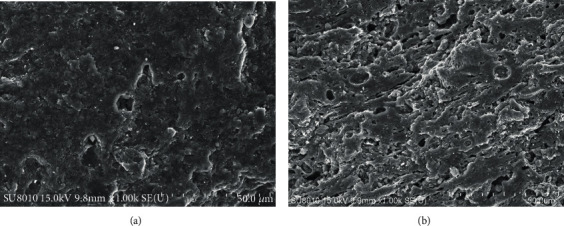
SEM image of OS. (a) Before catalytic pyrolysis. (b) After catalytic pyrolysis.

Figure 6 shows the average yield of the main pyrolysis products of Fushun OS adding Cu_m_Ni_n_/SA. The final pyrolysis temperature was steadily controlled at 520°C. Figure 6 also shows that SC was the main pyrolysis product, accounting for more than 76.1%, which corresponds to the high ash content of Fushun OS. The addition of Cu_m_Ni_n_/SA increased the yield of SC and OS and reduced the yield of exhaust gas, because Cu_m_Ni_n_/SA had changed the chemical constitution and physical structure in the inner of OS during the pyrolysis process. As shown in Figure 7, before the catalytic pyrolysis of OS, the surface structure is compact with only a few small pits (Figure 7(a)); after the catalytic pyrolysis, the surface structure of OS becomes obviously porous and loose (Figure 7(b)). The coke yield increased because higher pyrolysis temperature is conducive for the dissociation of the massive organic matter and their release from the shale, leaving a portion of organic matter being carbonized before separating from the shale [21, 22]. Moreover, such a reaction was enhanced owing to the presence of Cu_m_Ni_n_/SA. Some organic matter, in the meanwhile, combined with metal elements and formed intermediate products, together with which together with coke would adhere to the inner walls of shale pores, also leading to the pore blockage and strengthening the coking reaction [23]. Considering the increase of the SO yield, OS-Cu_0_Ni_1_/SA and OS-Cu_2_Ni_1_/SA showed stronger capabilities to a higher oil yield with an increase of 3.5% and 3.1%, respectively.

### 3.3. Component Analysis

#### 3.3.1. Semicoke ATR-FTIR Analysis

**Figure 8:**
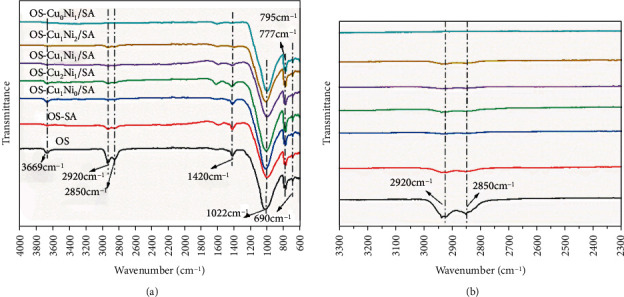
ATR-FTIR spectra of OS and pyrolytic SC.

Figure 8(a) shows the ATR-FTIR spectrum of the mixed pyrolysis product SC of OS after adding SA and Cu_m_Ni_n_/SA, and Figure 8(b) is the spectrum with the wavenumber of 2300~3300, showing a roughly the same trend of the spectra of several samples. Inorganic minerals were mainly silicate (1022 cm^−1^), quartz (690 cm^−1^, 795 cm^−1^, 777 cm^−1^), carbonate calcite (1420 cm^−1^), and silicate kaolinite (3669 cm^−1^). The spectrum should have included the tensile and flexural vibrations of the aliphatic and aromatic groups of kerogen, but these vibrations overlapped with the peaks of minerals such as carbonate, quartz, and clay [15]. The most obvious absorption peaks-the characteristic absorption peaks of aliphatic hydrocarbons of organic matter were located at 2920 cm^−1^ and 2850 cm^−1^ and were related to aliphatic CH bonds. Therefore, the main component of organic matter in OS was aliphatic hydrocarbons [24, 25]. After the catalytic pyrolysis of OS, the characteristic absorption peaks of the SA spectrum at 2920 cm^−1^ and 2850 cm^−1^ appeared obviously lower, indicating that the organic matter in the OS was separated during the pyrolysis process. As it is shown in Figure 8(b), the addition of Cu_m_Ni_n_/SA promoted almost complete pyrolysis of organic matter in OS.

#### 3.3.2. ATR-FTIR Analysis of Shale Oil

**Figure 9:**
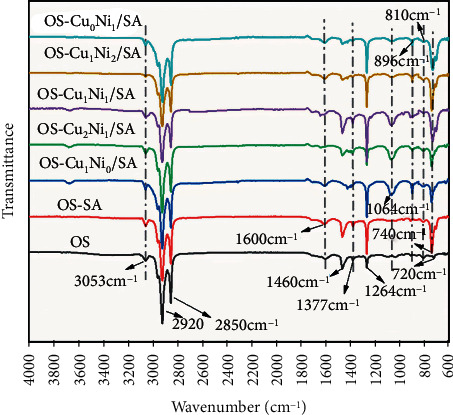
ATR-FTIR of mixed pyrolysis product SO of OS under SA and Cu_m_Ni_n_/SA.

Figure 9 shows the ATR-FTIR spectrum of the mixed pyrolysis product SO of OS under SA and Cu_m_Ni_n_/SA, demonstrating an overall identical trend of the spectra of different samples, the main functional groups fluctuating within the wavenumber region of 3000~2800 cm^−1^, 1600~1000 cm^−1^, and 900~700 cm^−1^. The wavenumber 3000~2800 cm^−1^ mainly corresponded to aliphatic substances and the two most obvious peaks of OS located in this range, mainly near 2920 cm^−1^ and 2850 cm^−1^, which corresponded to aliphatic methylene groups (CH_2_) C-H asymmetric and symmetrical vibration [12, 26, 27]. The strong peak at 1460 cm^−1^ was due to the asymmetric bending of the CH_3_ and CH_2_ groups, and the peak at 1377 cm^−1^ appeared rather weak because of the symmetric bending of CH_3_, which proved the existence of aliphatics [27]. The addition of SA and Cu_m_Ni_n_/SA may promote the formation of olefins. It is generally believed that when the wavenumber is greater than 3000 cm^−1^, the vibration is caused by the stretching of the CH bond of ethylene or aromatic groups. The distinct shoulder peak near 3053 cm^−1^ reveals the presence of unsaturated compounds, corresponding to the vibration of *ν*(*C*_*sp*^2^_ − *H*). The characteristic absorption peak at 896 cm^−1^ may represent the out-of-plane bending vibration of the alkene CH (R_1_R_2_C=CH_2_) [28, 29]. In addition, there is a small characteristic peak of bending vibration at about 810 cm^−1^ of the SO of the seven samples. Davis et al. believes that the peak at 810 cm^−1^ is an aromatic C–H out-of-plane bending mode, proving the existence of aromatic compounds in SO [30]. The broadened peak group of 1600 cm^−1^ may represent the aromatic ring C=C group and oxygen-containing functional group stretching vibration. Chi believes that [31], due to the oxygen-containing functional group connected to the aromatic ring, this peak group enhances the nuclear vibration of the aromatic ring. The characteristic absorption peak near 1264 cm^−1^ may represent the tensile vibration of the aromatic ether C-O, indicating that the addition of SA and Cu_m_Ni_n_/SA may enhance the formation of aromatic compounds [32]. The characteristic peak near 1064 cm^−1^ may be the characteristic C-O stretching absorption peak of alcohol, phenol, and ester [33]. In addition, it can be found that there is a characteristic peak of 720 cm^−1^ long chains in the FTIR spectrum of the single OS pyrolysis. Such a small peak was attributed to the swing vibration of the long methylene chains (-CH_2_-), which indicated that there was a methylene aliphatic chains with chain lengths greater than 4 during the direct OS pyrolysis. However, after adding SA and Cu_m_Ni_n_/SA, the peak in the FTIR spectrum of SO disappeared and appeared a characteristic peak of short-chain hydrocarbons at 740 cm^−1^ [34], indicating that both SA and Cu_m_Ni_n_/SA are to a certain extent capable of promoting the decomposition of long-chain hydrocarbons to short-chain hydrocarbons.

The analysis shows that the main component of SO was aliphatic hydrocarbons and a small number of aromatic hydrocarbons. The addition of SA and Cu_m_Ni_n_/SA may not only promote the formation of aliphatic olefins and aromatic compounds in SO but also the decomposition of long-chain aliphatic hydrocarbons to short-chain aliphatic hydrocarbons. Therefore, SA and Cu_m_Ni_n_/SA changed the composition of SO during the OS pyrolysis, providing a basis for improving the quality of SO in the actual industry.

#### 3.3.3. GC-MS Analysis of Shale Oil

**Figure 10:**
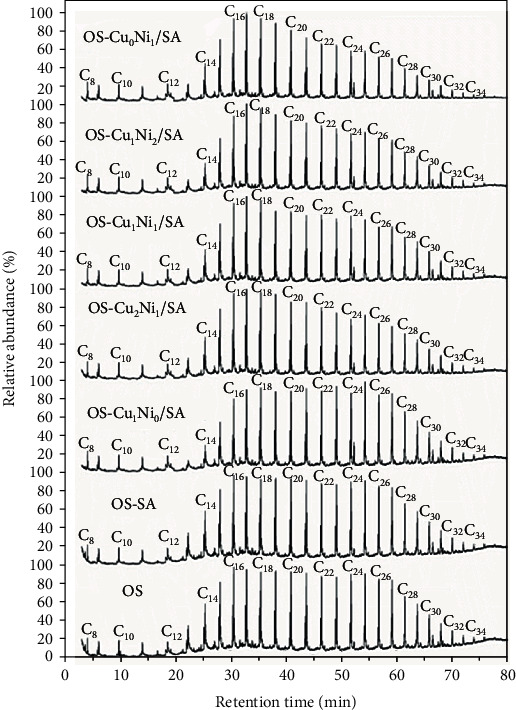
GC-MS total ion diagram of mixed pyrolysis product SO of OS under SA and Cu_m_Ni_n_/SA.

Figure 10 shows the GC-MS total ion diagram of the mixed pyrolysis product SO of OS under SA and Cu_m_Ni_n_/SA. As shown in the figure, all chromatograms were dominated by alkanes and alkenes with carbon numbers ranging from 8 to 34. Normal alkenes and normal alkanes with the same carbon number formed a double peak of aliphatic hydrocarbons, and aliphatic hydrocarbons with adjacent carbon numbers were distributed with branched alkanes, branched alkenes, aromatic hydrocarbons, and oxygen-containing compounds. Oxygen-containing compounds included acids, alcohols, esters, ketones, and phenols, and aromatic hydrocarbons included naphthalene, anthracene, benzene, and benzene series. The relative abundances of *n*-paraffins and *n*-alkenes first increased and then decreased as the carbon number increased and reached the maximum at C_16_~C_18_. These results were of high correspondence to the previous researchers' experimental results [14, 35].

**Figure 11:**
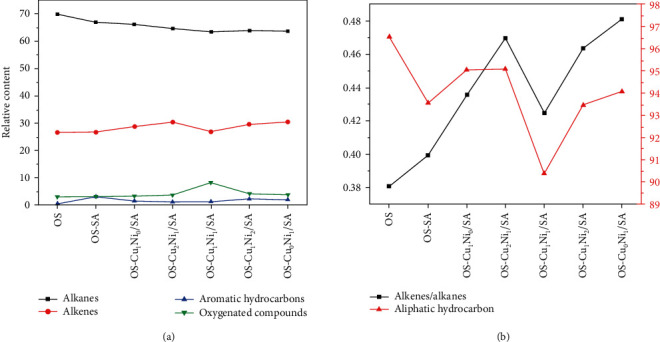
Information on four main components in SO of pyrolysis product. (a) Relative content. (b) Ratio of olefins to alkanes, aliphatic hydrocarbon content.

In order to estimate the influence of Cu_m_Ni_n_/SA on SO composition, the relative content of the main components was given by the ion peak area of GC-MS.  Figure 11(a) shows the content of the four main components in the pyrolysis product SO. With the change of the Cu-Ni metal salt mixing ratio, the content of each component in SO was slightly different, among which alkanes ranked the most. As the main component of SO, aliphatic compounds played an important role in improving the quality and quantity of SO. The content of alkanes and olefins in aliphatic hydrocarbons accounted for more than 90% of the relative content of the main components. The addition of SA and Cu_m_Ni_n_/SA reduced the content of alkanes and increased the content of olefins. Among them, the content of alkane in OS-Cu_1_Ni_1_/SA decreased the most, followed by OS-Cu_0_Ni_1_/SA; the content of olefin in OS-Cu_0_Ni_1_/SA increased the most, followed by OS-Cu_2_Ni_1_/SA, possibly because the secondary cracking reaction of SO led to a large number of normal types of paraffin to be transferred to cycloalkanes and alkenes. While the existence of Cu_m_Ni_n_/SA strengthened such reaction to a certain extent [36].  Figure 11(b) shows the olefin/alkane ratio. Ballice [37] applies the olefin/alkane ratio to evaluate the cracking reaction of aliphatic hydrocarbons. In his research, it has been shown that the cracking reaction proceeded through a free radical mechanism so that smaller linear alkanes and alkenes were obtained, increasing the alkene/alkane ratio. Therefore, SA and Cu_m_Ni_n_/SA in this study can catalyze the cracking of aliphatic hydrocarbons. The changes in the OS-Cu_0_Ni_1_/SA and OS-Cu_2_Ni_1_/SA samples are more obvious, indicating when Cu and Ni transition metal salt loading mass ratio is 0 : 1 and 2 : 1; it has a strong catalytic effect, which is consistent to thermodynamics results.

In addition, as shown in Figure 11(a), the content of aromatic hydrocarbons was affected by the following sequence: OS − SA > OS − Cu_1_Ni_2_/SA > OS − Cu_0_Ni_1_/SA > OS − Cu_1_Ni_0_/SA > OS − Cu_1_Ni_1_/SA > OS − Cu_2_Ni_1_/SA > OS, indicating that the presence of SA and Cu_m_Ni_n_/SA may catalyze the aromatization of aliphatic hydrocarbons to a certain extent and thus produce more aromatic hydrocarbons. The OS-SA samples produced the most aromatic hydrocarbons, which may be because the oil stayed so long time in the pores or on the surface of the OS and SA particles that the subsequent secondary cracking reaction was promoted. Burnham [38] believes that SO cracking occurs through two processes: the coking of hydrogen-depleted materials and the cracking of aliphatic splitting into gases. The reasons for the formation of aromatic hydrocarbons through secondary reactions may include gas-phase cracking of aliphatic compounds or through Diels-Alder type reaction [38–41]. The addition of Cu_m_Ni_n_/SA may promote the formation of aromatic hydrocarbons to a certain extent because of the abovementioned secondary reactions on the one hand and the low molecular weight (LMW) alkanes and alkenes produced by the cracking of large aliphatic hydrocarbons on the other. The catalysis of strong acidic sites of metal salt cyclized LMW olefins and dienes to form cycloalkenes, and cycloalkanes were dehydrogenated to produce aromatics [39]. However, the sample with Cu_m_Ni_n_/SA added in this study produced less aromatic hydrocarbons than the sample with SA only. This may be due to the coking reaction of hydrogen-depleted substances during the diffusion in the pyrolysis furnace with the increase of temperature made aromatic hydrocarbon tend to coke before volatilization, forming solid products or coke [42–44]. The presence of Cu_m_Ni_n_/SA significantly increased the probabilities of the above coking reaction, leading to a reduction of the relative content of aromatic hydrocarbons [14, 45, 46]. The presence of aromatic hydrocarbons weakened the stability of SO and is of toxicity in use [38, 47]. Therefore, based on the stability of SO and safety in use, the order of preference is ranked as OS − Cu_2_Ni_1_/SA > OS − Cu_1_Ni_1_/SA > OS − Cu_1_Ni_0_/SA > OS − Cu_0_Ni_1_/SA > OS − Cu_1_Ni_2_/SA > OS − SA. In addition, the presence of SA and Cu_m_Ni_n_/SA promoted the formation of not only aromatic compounds but also oxygen-containing compounds, with OS-Cu_1_Ni_1_/SA containing the most oxygen-containing compounds.

**Figure 12:**
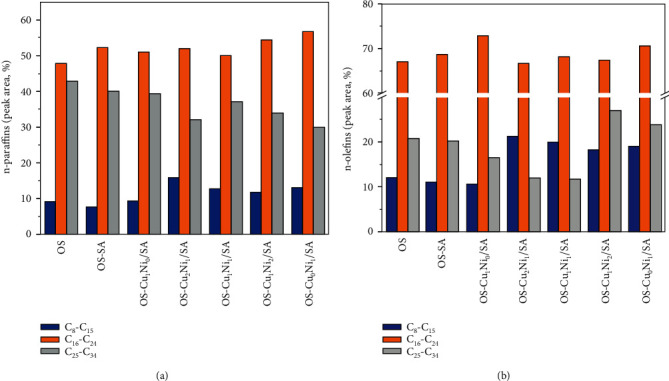
Distribution of *n*-paraffins and *n*-olefins in SO: (a) *n*-paraffins, (b) *n*-olefins.

According to the GC-MS analysis of SO, *n*-paraffins and *n*-alkenes are the main components in SO samples, which can be further divided into the following categories according to the number of carbon atoms: C_8_~C_15_, C_16_~C_24_, and C_25_~C_34_. The content of these components based on peak area was normalized to 100%.  Figure 12 shows the relative peak areas of different samples at different carbon numbers. The figure shows that the addition of SA and Cu_m_Ni_n_/SA decreased the content of C_25_-C_34_ heavy alkanes and olefins and increased the content of C_8_~C_15_ and C_16_~C_24_ light hydrocarbons. This result indicates that SA and Cu_m_Ni_n_/SA can promote the decomposition tendency of heavy oil fractions to light oil, possibly because the cracking reaction of gas-phase oil is promoted after the SA and SA load transition mental salts so that long-chain aliphatic compounds are converted into short-chain hydrocarbons. In this process, long-chain normal types of paraffin were cracked and formed short-chain alkanes, alkenes, and cycloalkanes, resulting in increasing the content of shorter-chain hydrocarbons. This also firmly proves the Cu_m_Ni_n_/SA catalyzes aliphatic pyrolysis, which is consistent with the results of ATR-FTIR analysis [36]. In addition, compared with the addition of SA pyrolysis, the content of aliphatic hydrocarbons in the sample with Cu_m_Ni_n_/SA changed significantly, indicating the strong catalytic activity of SA after being loaded with Cu-Ni double transition metal salt. Therefore, Cu_m_Ni_n_/SA can significantly affect the composition of SO. Among them, the contents of *n*-paraffins and *n*-alkenes in OS-Cu_2_Ni_1_/SA and OS-Cu_0_Ni_1_/SA samples changed the most, that is, the catalytic effect performed better when the Cu-Ni loading mass ratio was 2 : 1 and 0 : 1.

## 4. Conclusion

After analyzing the pyrolysis characteristics of OS containing a different ratio of Cu to Ni with TG-MS technology method then pyrolysis product SO under methods including FTIR and GC-MS, the conclusions are presented as follows:

1. The effects of SA and Cu_m_Ni_n_/SA on the pyrolysis characteristics of Fushun OS were investigated by thermogravimetry. It is found that the addition of SA and Cu_m_Ni_n_/SA has different effects on the pyrolysis behavior of OS. The total mass loss rate is ranked as OS < OS − SA < OS − Cu_m_Ni_n_/SA; the existence of SA and Cu_m_Ni_n_/SA reduces the initial OS Pyrolysis temperature, termination temperature, maximum weight loss rate, and its corresponding temperature (*T*_max_). Cu_m_Ni_n_/SA falls further, and the apparent activation energy, combined with kinetic analysis, is ranked as OS > OS − SA > OS − Cu_m_Ni_n_/SA, indicating that the existence of SA promotes the pyrolysis of OS to a certain extent, but the catalytic effect is weaker to that of Cu_m_Ni_n_/SA. OS-Cu_0_Ni_1_/SA, OS-Cu_2_Ni_1_/SA, and OS-Cu_1_Ni_1_/SA in Cu_m_Ni_n_/SA strengthened the catalytic effects, and *T*_max_ decreased by 12.9°C, 4.0°C, and 3.6°C, respectively, and apparent activation energy decreased by 35.2%., 33.9%, and 29.6%. It was also found that the two transition metal salts supported by Cu_m_Ni_n_ may have a complicated effect during the OS pyrolysis process, rather than catalyzing the OS pyrolysis reaction in their respective ranges
2. The products of SA-, Cu_m_Ni_n_/SA-, and OS-mixed pyrolysis were analyzed by the pyrolysis device. It is found that the main product of OS pyrolysis is SC, accounting for more than 76.1%; the addition of Cu_m_Ni_n_/SA promotes the coking reaction of OS pyrolysis, leading to an increase in the yield of SC. The existence of Cu_m_Ni_n_/SA also increases the yield of SO and reduces the emissions yield. Among them, OS-Cu_0_Ni_1_/SA and OS-Cu_2_Ni_1_/SA further increase the SO yield by 3.5% and 3.1%, respectively
3. The analysis of pyrolysis products SC and SO with the FTIR method shows that the inorganic minerals in SC are mainly composed of silicate, quartz, carbonate calcite, etc.; the main component of organic matter in SO is aliphatic hydrocarbons, a small number of aromatic hydrocarbons, and oxygen-containing compounds as well. The existence of SA and Cu_m_Ni_n_/SA promotes OS pyrolysis secondary cracking, aromatization, and other relevant reactions, forming olefins and aromatic compounds in SO and promoting the decomposition of long-chain aliphatic hydrocarbons to short-chain aliphatic hydrocarbons
4. The effects of SA and Cu_m_Ni_n_/SA on different components of SO were further studied by GC-MS. SO is complex and diverse in its composition, mainly dominated by alkanes and alkenes with carbon atoms of 8 to 34; alkanes, alkenes, oxygenates, and aromatics are the main components in SO, with alkanes and alkenes being the majority, accounting for more than 90% of its relative content. The addition of SA and Cu_m_Ni_n_/SA reduces the content of alkanes and increases that of olefins. Among them, the content of alkane in OS-Cu_1_Ni_1_/SA decreases the most, followed by OS-Cu_0_Ni_1_/SA. The content of olefin in OS-Cu_0_Ni_1_/SA increases the most, followed by OS-Cu_2_Ni_1_/SA. The presence of SA and Cu_m_Ni_n/_SA may also catalyze the aromatization of aliphatic hydrocarbons to a certain extent, increasing the production of aromatic hydrocarbons. The presence of Cu_m_Ni_n_/SA may catalyze aromatization, but at the same time, it also aggravates the coking reaction of hydrogen-poor substances, resulting in Cu_m_Ni_n_/SA catalytic pyrolysis producing less aromatic hydrocarbons than SA. The aromatic hydrocarbon content is ranked as follows: OS − SA > OS − Cu_1_Ni_2_/SA > OS − Cu_0_Ni_1_/SA > OS − Cu_1_Ni_0_/SA > OS − Cu_1_Ni_1_/SA > OS − Cu_2_Ni_1_/SA > OS. In addition, the presence of SA and Cu_m_Ni_n_/SA also promotes the formation of oxygen-containing compounds, and OS-Cu_1_Ni_1_/SA has the largest content of oxygen compounds
5. OS-Cu_0_Ni_1_/SA and OS-Cu_2_Ni_1_/SA promote catalysis and effectively increase the SO yield. However, OS-Cu_0_Ni_1_/SA produces more aromatic hydrocarbons, which weakens the stability of SO and is of toxicity in use

Considering comprehensively the catalytic effect, SO yield, and SO stability, this paper suggests that the Cu-Ni/shale ash-based dual transition metal catalyst Cu-Ni loading ratio preferably be 2 : 1.

## Figures and Tables

**Table 1:** Industrial analysis and elemental analysis of oil shale (OS) and shale ash (SA).

Industrial analysis, %					Elemental analysis, %				
Sample	*M_ad_*	*A_ad_*	*V_ad_*	*FC_ad_*∗	*C_ad_*	*H_ad_*	*N_ad_*	*O_ad_* ^∗^	*S_ad_*
OS	2.86	77.29	17.77	2.08	10.91	1.82	0.78	5.91	0.33
SA	0.48	90.68	5.96	2.88	5.16	0.56	1.06	0.64	0.83

^∗^subtraction method.

**Table 2:** Ash XRF (X-ray fluorescence) analysis of OS and SA.

Constituent, wt %	SiO_2_	Al_2_O_3_	Fe_2_O_3_	K_2_O	TiO_2_	MgO	P_2_O_5_	SO_3_	CaO	Other
OS	40.48	13.30	9.78	1.01	0.89	0.88	0.81	0.80	0.79	31.26
SA	58.15	23.18	10.26	1.45	1.35	1.42	1.25	1.53	1.08	0.33

**Table 3:** Comparison among thermogravimetric data of different samples.

Samples	Starting temperature/°C	Termination temperature/°C	Maximum weight loss rate /%/min	Temperature at maximum weight loss rate/°C	Sample weight loss rate/wt.%		
					Room temperature~200°C	200~600°C	600~900°C
OS	418.1	520.8	1.609	471.2	0.10	15.07	3.15
OS-SA	413.7	517.4	1.654	470.4	0.11	15.36	3.55
OS-Cu_1_Ni_0_/SA	396.5	520.2	1.420	469.0	0.44	16.12	3.99
OS-Cu_2_Ni_1_/SA	392.9	519.2	1.589	467.2	0.55	17.05	3.83
OS-Cu_1_Ni_1_/SA	398.0	520.0	1.517	467.6	0.20	16.98	3.47
OS-Cu_1_Ni_2_/SA	401.8	520.3	1.499	468.0	0.33	16.14	3.85
OS-Cu_0_Ni_1_/SA	394.5	515.7	1.586	458.3	0.59	17.35	4.17

**Table 4:** Pyrolysis kinetic parameters calculated via Coats-Redfern method.

Samples	Temperature/°C	Activation ability/kJ/mol	Pre-reference factor/min^−1^	Correlation coefficient (*R*^2^)
OS	418.1-520.8	39.2	2.6 × 10^3^	0.9832
OS-SA	413.7-517.4	36.6	1.3 × 10^3^	0.9805
OS-Cu_1_Ni_0_/SA	392.9-519.2	26.3	80.3	0.9835
OS-Cu_2_Ni_1_/SA	399.5-520.2	25.9	58.0	0.9874
OS-Cu_1_Ni_1_/SA	398.0-520.0	27.6	113.3	0.9904
OS-Cu_1_Ni_2_/SA	401.8-520.3	28.8	155.3	0.9899
OS-Cu_0_Ni_1_/SA	392.5-515.7	25.4	109.7	0.9917

## Data Availability

All the data used to support the findings of this study are included within the article.
